# Bowel Dysfunctions in Young Adults with Multiple Sclerosis: A Retrospective Study

**DOI:** 10.3390/medsci13030123

**Published:** 2025-08-11

**Authors:** Edoardo Sessa, Lilla Bonanno, Carla Susinna, Daniela Ivaldi, Gabriele Triolo, Roberta Lombardo, Giangaetano D’Aleo, Carmela Rifici, Viviana Lo Buono

**Affiliations:** IRCCS Centro Neurolesi Bonino Pulejo, 98123 Messina, Italy; edoardo.sessa@irccsme.it (E.S.); lilla.bonanno@irccsme.it (L.B.); daniela.ivaldi@irccsme.it (D.I.); gabriele.triolo@irccsme.it (G.T.); roberta.lombardo@irccsme.it (R.L.); giangaetano.daleo@irccsme.it (G.D.); carmela.rifici@irccsme.it (C.R.); viviana.lobuono@irccsme.it (V.L.B.)

**Keywords:** multiple sclerosis, bowel dysfunction, quality of life, young adults, fecal incontinence, constipation

## Abstract

Background/Objectives: Multiple sclerosis (MS) is a chronic neurodegenerative disorder mainly affecting young adults and can greatly impair quality of life (QoL). Among its often overlooked but significant symptoms are bowel dysfunctions (BD), such as constipation and fecal incontinence, which can impact physical, emotional, and social well-being, especially in younger patients. This study aims to investigate the impact of BD on the QoL in young adults diagnosed with relapsing-remitting MS (RRMS) and mild disability. Methods: This retrospective cross-sectional study examined the effect of BD on QoL in 110 young adults with RRMS and mild disability (EDSS ≤ 3.5). Bowel symptoms were assessed using the Wexner Incontinence and Constipation Scales, while QoL was measured with the MSQoL-54 questionnaire. Statistical analyses were performed to examine correlations between BD severity and QoL domains. Results: Our findings showed significant correlations between the severity of intestinal symptoms and different domains of QoL, like physical functioning, emotional well-being, and social functioning. Abdominal pain and liquid fecal incontinence were especially linked to lower mental and physical health scores. Subgroup analyses also indicated gender-specific vulnerabilities, with women showing distinct effects on social and emotional dimensions. Conclusion: BD represents an important burden on bowel dysfunctions for young people with MS, deeply impacting various dimensions of QoL. This underscores an urgent need for an integrated, multidisciplinary care model that tackles physical symptoms but also psychological and social challenges. A holistic clinical strategy is vital to improving the overall well-being of this population.

## 1. Introduction

Multiple sclerosis (MS) is a chronic neurological disease caused by an autoimmune and inflammatory neurodegenerative process that affects the central nervous system (CNS) [1]. In young adults, it is one of the primary causes of neurologic impairment.

Although the exact etiology of MS remains unknown, current evidence suggests that its onset results from a complex interaction involving genetic susceptibility, viral agents, nutritional and metabolic imbalances, environmental triggers, and lifestyle-related factors [2].

Clinical symptoms of the disease appear between the ages of 20 and 40. This period of early to mid-adulthood is not only critical for personal and professional development but also represents a phase characterised by heightened sexual activity and reproductive planning [3]. Myelin loss and axonal injury are the primary pathological markers of the disease [4]. Clinically, MS manifests with spasticity, fatigue, ataxia, tremors, bladder dysfunction, pain, optic neuritis, and cognitive impairment during the disease [5]. Other common symptoms in people with MS include bowel dysfunction (BD), which can manifest as constipation and/or fecal incontinence [6]. Constipation involves infrequent or difficult bowel movements and may be accompanied by symptoms like tiredness, bloating, and abdominal pain. Fecal incontinence is the involuntary loss of stools or flatus for at least 1 month. This can occur without the patient being aware of it, or it can be accompanied by a variable degree of urgency. These symptoms often result from a combination of slowed intestinal transit, impaired anorectal coordination, reduced abdominal and pelvic floor muscle function, and decreased sensation of rectal fullness [7].

The neural system plays a complex role in controlling bowel function, especially through enteric and autonomic pathways that combine inputs from the stomach, spinal cord, and brain [8]. BD is often linked to axonal damage or demyelination that disrupts neuronal communication in MS. These pathological alterations can affect the coordinated reflexes required for normal bowel motility and sphincter control, particularly within spinal cord circuits involved in autonomic modulation [9].

However, similar to other specific symptoms in MS, BD does not always correlate with MRI lesions. This clinical-radiological dissociation suggests that additional mechanisms may contribute to bowel dysfunction beyond structural damage. One emerging hypothesis is that circulating toxic factors, such as proinflammatory cytokines, autoantibodies, or other immune mediators, may exert deleterious effects on neuronal or glial function in a way not easily detectable via conventional imaging techniques [10]. These factors may modify neurotransmission, disrupt the blood-brain barrier, or interfere with gut-brain signalling, thereby contributing to autonomic dysfunction [11]. BD in people with MS is frequently linked to higher levels of disability and longer disease duration. However, it can also affect subjects with mild disability and a recent diagnosis. In certain cases, BD may even be one of the initial clinical manifestations of the disease [12].

BD is a significant symptom that can negatively impact patients’ overall well-being, interpersonal relationships quality of life (QoL) [13].

BD is a significant yet often under-recognised symptom of MS [14]. BD may appear at any stage of the MS and tends to worsen as the disease progresses. For young subjects with MS, bowel symptoms can lead to embarrassment, social withdrawal, and difficulties in maintaining intimate relationships, further impacting psychological well-being. BD manifests in both men and women and can occur at any stage of the disease, though it tends to be more pronounced as the disease progresses. The psychosocial impact of these disturbances is particularly challenging for young people with MS. The emotional burden of MS-related bowel problems can be exacerbated by societal stigma surrounding disability and sexual health [15].

This study aimed to assess the impact of BD on QoL in young adults living with MS.

## 2. Materials and Methods

### 2.1. Study Design

This retrospective cross-sectional cohort study involved young adults with MS who attended the Neurology outpatient clinic at the Istituto di Ricovero e Cura a Carattere Scientifico (IRCCS) Centro Neurolesi “Bonino Pulejo” of Messina between April and June 2023. Informed consent was obtained from all participants by the Declaration of Helsinki.

The study was approved by the Ethics Committee of IRCCS Centro Neurolesi Bonino Pulejo: E147/22 del 19.10.22.

### 2.2. Study Population

Young adults with relapsing-remitting multiple sclerosis (RRMS) were consecutively recruited.

MS was diagnosed by three experienced neurologists with the 2017 revised McDonald criteria. The Expanded Disability Status Scale (EDSS) [16] was used to evaluate patients’ disability.

Inclusion criteria: diagnosis of RRMS confirmed through clinical history and MRI findings; absence of progressive disease signs on MRI or clinical evaluation for at least one year before enrollment; age between 18 and 35 years old; RR disease course; EDSS ≤3.5; Montreal Cognitive Assessment (MoCA) score > 25; participants must provide written informed consent before participating in the research agreeing to all study procedures.

Exclusion criteria: presence of major psychiatric disorders; other neurological diseases; pathologies in comorbidity; use of antidepressant or psychotropic medications.

### 2.3. Outcome Measures

The primary outcome of this study was the prevalence of BD, evaluated using two validated instruments: the Wexner Incontinence Scale (WexInc) and the Wexner Constipation Scale (WexCon).

The WexInc scale consists of five multiple-choice questions scored from 0 (never) to 4 (daily). The scale evaluates the type of incontinence (solid or liquid stool), the occurrence of gas, any lifestyle modifications, and the use of protective pads [17].

The WexCon assesses eight factors with scoring ranges from 0 to 32: bowel movement frequency, painful defecation, sensation of incomplete evacuation, abdominal pain, need for assistance, duration of constipation, time spent during attempts, unsuccessful evacuation attempts per 24 h [18].

Secondary outcomes included measures of QoL, which were evaluated using the Italian version of the Multiple Sclerosis QoL-54 (MSQoL-54) [19]. This scale provides a comprehensive evaluation of health-related quality of life by seamlessly combining generic measures with MS-specific components into a single, cohesive instrument.

The54-item instrument generates 12 subscales: Physical Function (PF), Role Limitations Physical (RLP), Role Limitations Emotional (RLE), Pain (P), Emotional Well-Being (EWB), Energy (E), Health Perceptions (HP), Social Function (SF), Health Distress (HD), Overall Quality of Life (OQoL), Cognitive function (CF), Sexual Function (Sex F). MSQoL-54 produces two additional summary composite scores: physical health and mental health scores.

All participants underwent neuropsychological evaluation as part of routine clinical follow-up, administered by two trained neuropsychologists.

### 2.4. Statistical Analysis

Descriptive statistical methods were employed to examine and summarize the sociodemographic characteristics of the sample. The Shapiro-Wilk test was applied to assess the normality of the distribution of variables. Continuous variables were expressed as mean ± standard deviation for data with a normal distribution.

In contrast, variables that did not follow a normal distribution were summarised using the median and interquartile range (i.e., the first to third quartile). Spearman’s rank correlation coefficients were computed to assess associations between disease duration (DD), functional domains, and bowel symptoms across the entire sample. Subsequently, the dataset was stratified by sex (male and female), and further analyses were performed. Between-group comparisons were conducted to assess sex differences in bowel-related symptoms and functional domain scores, using either the Student’s unpaired *t*-test or the Mann–Whitney U test, depending on the normality of the data.

Within-group correlation analyses were also carried out separately for males and females to explore potential sex-specific patterns of association, using Spearman’s correlation. The strength of correlation coefficients (r) was interpreted as follows: values between 0.10 and 0.29 were considered small, between 0.30 and 0.49 moderate, and ≥0.50 large, in line with conventional guidelines.

Due to the exploratory nature of the study and the relatively small sample size, the Benjamini–Hochberg false discovery rate (FDR) correction was applied to control for multiple comparisons across all correlation analyses. However, as most associations did not remain significant after correction, we report uncorrected *p*-values in the tables. Results with *p*-values between 0.05 and 0.07 are described as “statistical trends”. Subgroup analyses by sex were also exploratory and not formally powered to detect small effects. The open-source R software (version 4.2.2) developed by the R Foundation for Statistical Computing, Vienna, Austria, was used for statistical analyses.

A confidence level of 95% was established with a 5% alpha error. Statistical significance was determined at *p* < 0.05 (two-tailed); Results with *p*-values between 0.05 and 0.07 were not considered statistically significant but are reported as descriptive trends, suggesting potential associations that may warrant further investigation.

## 3. Results

### 3.1. Correlations Between Functional Domains and Bowel Dysfunction Symptoms

A total of 110 young adults with RRMS were included in the analyses. Characteristics of the sample were presented in Table 1.

Spearman correlation analyses revealed several significant relationships between functional domains and bowel symptoms. SF was significantly associated with multiple aspects of constipation; it correlated positively with the time required for defecation (rho = 0.26, *p* = 0.01), abdominal pain (rho = 0.24, *p* = 0.01), and the total constipation score (rho = 0.20, *p* = 0.03). Additionally, it showed a trend-level positive correlation with defecation difficulty (r = 0.19, *p* = 0.05), and a trend toward a negative correlation with solid stool leakage (r = −0.19, *p* = 0.05). Significant associations also emerged for Ewb. This domain was positively correlated with the time required for defecation (r = 0.21, *p* = 0.03) and the total constipation score (r = 0.19, *p* = 0.05) while showing a negative correlation with the presence of liquid stool leakage (r = −0.27, *p* = 0.00). A similar pattern was observed for TOT MH, which correlated positively with the duration of constipation (r = 0.21, *p* = 0.03), time required for defecation (r = 0.205, *p* = 0.03), and total constipation score (r = 0.21, *p* = 0.03). Several trend-level correlations were identified across other domains. PF showed a positive trend with incomplete defecation (r = 0.17, *p* = 0.08). HP was also positively associated at the trend level with time required for defecation (r = 0.183, *p* = 0.06). Pain demonstrated trends with duration of constipation (r = 0.16, *p* = 0.09), total constipation score (r = 0.169, *p* = 0.08), and total incontinence (r = 0.16, *p* = 0.09). Trend-level associations were further observed for HD PH with time required for defecation (r = 0.17, *p* = 0.08), HD MH with liquid stool leakage (r = −0.17, *p* = 0.08), TOT PH with time required for defecation (r = 0.19, *p* = 0.04) and abdominal pain (r = 0.16, *p* = 0.09), and Cognitive Functioning (CF) with both duration of constipation (r = 0.19, *p* = 0.05) and total constipation score (r = 0.19, *p* = 0.05). Finally, RIE showed a trend with time required for defecation (r = 0.16, *p* = 0.09), and Overall Quality of Life (OQoL) showed a negative trend with diaper use (r = −0.16, *p* = 0.10).

### 3.2. Subgroup-Level Associations Between Bowel Symptoms and Health Domains

Between-group comparisons revealed no statistically significant differences (Table 2 and Table 3). Within-group analyses showed several significant correlations. In the male sample, we showed a negative correlation between HP and DD (r = −0.38; *p* = 0.03); incomplete defecation was positively correlated with RLP (r = 0.37, *p* = 0.03) and RLE (r = 0.36, *p* = 0.03). Constipation duration was positively correlated with PH (r = 0.35, *p* = 0.04), RLP (r = 0.39, *p* = 0.02), and RLE (r = 0.38, *p* = 0.02). Trends were also observed between constipation duration and Energy (r = 0.30, *p* = 0.08) and SF (r = 0.33, *p* = 0.06). The time required for defecation showed positive correlations with HD and MH (r = 0.35, *p* = 0.04), EWB (r = 0.44, *p* = 0.01), RLE (r = 0.38, *p* = 0.03), and TOT MH (r = 0.37, *p* = 0.03), with trends for RLP (r = 0.31, *p* = 0.08) HD and PH (r = 0.34, *p* = 0.05). Unsuccessful defecation attempts were significantly associated with RLP (r = 0.37, *p* = 0.03) and RLE (r = 0.44, *p* = 0.01). Abdominal pain showed positive correlations with PF (r = 0.50, *p* < 0.001), RLP (r = 0.42, *p* < 0.001), SF (r = 0.40, *p* = 0.02), Tot PH (r = 0.38, *p* = 0.02), and RLE (r = 0.47, *p* < 0.001). Total constipation scores were positively correlated with RLP (r = 0.38, *p* = 0.03) and RLE (r = 0.41, *p* = 0.01). A significant negative correlation was found between liquid fecal incontinence and both EWB (r = −0.49, *p* < 0.001) and TOT MH (r = −0.36, *p* = 0.03), with trends also observed between liquid fecal incontinence with HD and PH (r = −0.32; *p* = 0.06), HD and MH (r = −0.33, *p* = 0.06) and OQoF (r = −0.34; *p* = 0.05), and between solid fecal incontinence and EWB (r = −0.32, *p* = 0.06). A significant negative correlation emerged between liquid fecal incontinence and both EWB (r = −0.49, *p* < 0.001) and TOT MH (r = −0.36, *p* = 0.03). Trends were also observed between liquid fecal incontinence and HD and PH (r = −0.32, *p* = 0.06), HD and MH (r = −0.33, *p* = 0.06), and OQoF (r = −0.34, *p* = 0.05). Additionally, a trend was noted between solid fecal incontinence and EWB (r = −0.32, *p* = 0.06).

In the female subgroup, a significant positive correlation was found between time required for defecation and SF (r = 0.24, *p* = 0.04), as well as between manual assistance and EWBb (r = 0.27, *p* = 0.02).

Trends were observed between incomplete defecation and CF (r = 0.21, *p* = 0.07), constipation duration and EWB (r = 0.22, *p* = 0.06), manual assistance and pain (r = 0.21;*p* = 0.08), abdominal pain and HP (r = 0.21, *p* = 0.07), total constipation and CF (r = 0.21, *p* = 0.07), and intestinal disturbances and EWB (r = 0.22, *p* = 0.06).

## 4. Discussion

The results indicate that intestinal symptoms are associated with multiple dimensions of QoL in young adults with MS. While the primary outcome of the study was the prevalence of BD, the observed associations with QoL domains provide important insights into the broader impact of these symptoms. Notably, several strong correlations emerged between the severity of these symptoms and overall well-being. These associations suggest that both the persistence and intensity of symptoms may be linked to a broader decline in multiple QoL domains.

Abdominal pain, strongly linked to lower scores in both physical functioning and mental health, may represent an important factor contributing to daily life challenges in in these young individuals with MS. Furthermore, the significant negative correlations between liquid fecal incontinence and emotional well-being and mental health suggests that episodes of incontinence may be associated with considerable distress and limitations in daily functioning. Incomplete defecation showed significant positive correlations with rectal irritability parameters, suggesting that patients experiencing a sensation of incomplete evacuation tend to report increased rectal sensitivity and irritability. Similarly, longer constipation duration was associated with pelvic floor dysfunction, rectal irritability, and irritability of the external environment, implying that chronicity may exacerbate both physical and emotional symptoms.

The trends observed in the female subgroup suggest that BD is subtly intertwined with emotional, cognitive, and social dimensions, even when the symptoms are not overtly severe. These sex-specific trends, although exploratory and uncorrected for multiplicity, may point to subtle differences that warrant confirmation in larger, balanced cohorts. Given the small and unbalanced sample, these subgroup results should be interpreted with caution and are not intended to support definitive conclusions. Notably, the association between manual assistance and emotional well-being could imply that the use of compensatory strategies, although clinically necessary, may be experienced as burdensome or emotionally taxing in this group. In contrast, the overall sample exhibited stronger and more numerous correlations between physical symptoms (e.g., incomplete defecation, duration, unsuccessful attempts, abdominal pain) and physical/role limitations and emotional health, possibly reflecting a more generalized biopsychosocial impact.

BD iscommon in MS, and can range from incontinence or constipation to more severe complications. BD may appear early in the disease course and often persists as MS progression [20]. In young adults with MS, intestinal disorders are not merely physical ailments but have profound, multidimensional effects on various dimensions of well-being [21]. BD can negatively impact social interactions due to the necessity to organize daily life activities around bowel management [22]. These symptoms are also recognized as a contributing factor to reduced work performance and occupational limitations [23]. Furthermore, the persistent nature of BD can compromise the emotional state by causing the onset of anxiety and depression [24].

Proper management of BD should involve a multidisciplinary approach to improve QoL, including medications, lifestyle modifications, and, if necessary, advanced or mechanical interventions such as sacral nerve stimulation, transanal irrigation, or surgical procedures [25].

Clinicians should be aware of the multifaceted nature of chronic constipation, recognizing that physical symptoms often coexist with psychological distress. This suggests the importance of adopting a multidisciplinary approach that includes not only gastroenterological management but also psychological support. In particular, addressing abdominal pain and fecal incontinence should be prioritized due to their significant impact on both physical health and emotional well-being.

Several limitations in this study could affect the accuracy and applicability of its conclusions. First, the retrospective cross-sectional design limits the ability to establish causal relationships or observe changes over time. As such, temporal associations between bowel dysfunction and disease progression cannot be fully elucidated. A prospective longitudinal design would be more appropriate to assess the dynamic evolution of bowel symptoms throughout MS, enabling more robust conclusions about potential predictive factors and the long-term impact of bowel dysfunction. Additionally, implementing a longitudinal approach would offer more comprehensive insights into how these symptoms develop and interact with both physical and psychological aspects of the disease over time. In addition, participants did not undergo a standardized gastroenterological diagnostic work-up. As such, we cannot exclude the presence of primary gastrointestinal conditions that might contribute to or confound the reported bowel symptoms. This lack of objective clinical confirmation limits the specificity of our findings. Another significant limitation of this study is the relatively small sample size, which restricts the ability to generalize the clinical findings. Finally, due to the exploratory nature of this study and the relatively small sample size, results were interpreted using uncorrected *p*-values. While this increases the risk of false positives, strict correction procedures such as FDR may obscure potentially relevant patterns and are therefore not always required in hypothesis-generating research. Subgroup analyses by sex were performed for hypothesis-generating purposes only and were underpowered; therefore, these findings should be interpreted with caution. Despite these limitations, the study offers valuable preliminary evidence on the multidimensional burden of BD in a relatively understudied MS subpopulation.

## 5. Conclusions

This study highlights the significant and multidimensional impact of BD on the QoL of young adults with RRMS. The findings underscore that BD, particularly symptoms such as abdominal pain and fecal incontinence, is strongly associated with impairments across physical, emotional, and social domains. Even in MS subjects with minimal disability, these symptoms can contribute to marked distress and functional limitations. The observed gender-specific trends suggest that emotional and psychosocial burdens may be more pronounced in women, although further research with larger and more balanced samples is needed to confirm these findings.

Future research involving larger and more diverse samples is warranted to confirm the trends observed in this study and to deepen our understanding of the underlying mechanisms linking constipation-related symptoms to psychosocial outcomes in individuals with MS. In addition, longitudinal studies are warranted to better understand the evolution of BD over time and its interaction with disease progression. Addressing BD proactively could play a crucial role in improving overall health outcomes and daily functioning in people with MS.

Given the complex interplay between neurogenic BD and emotional, cognitive, and social well-being, such investigations are essential for developing comprehensive, patient-centered care strategies aimed at reducing the overall burden of disease and enhancing QoL in this population.

## Figures and Tables

**Table 1:** Demographic and clinical characteristics of the sample with RRSM.

Variable	All	Female	Males
Gender	110	75	35
Mean age (years)	30.5 ± 5.42	29.98 ± 5.41	31.64 ± 5.34
Mean education (years)	6.42 ± 2.53	10.48 ± 4.13	9.97 ± 3.55
Disease duration (years)	6.78 ± 2.12	6.66 ± 1.96	7.05 ± 2.44
EDSS score	2.9 ± 0.86	2.9 ± 0.97	2.9 ± 0.61

Legend: RRMS = Relapsing-Remitting Multiple Sclerosis; EDSS = Expanded Disability Status Scale.

**Table 2:** Wexner incontinence and constipation scores.

	All	Males	Females	*p*-Value
	Median (I–III Quartile)	Median (I–III Quartile)	Median (I–III Quartile)	
Frequency of bowel movements	0 (0–1.0)	0 (0–1.0)	0 (0–1.0)	0.64
Completeness: feeling of incomplete evacuation	2.0 (1.0–2.0)	1.0 (1.0–2.0)	2.0 (1.0–2.0)	0.19
Difficulty: painful evacuation efforts	2.0 (1.0–2.0)	1.5 (1.0–2.0)	2.0 (1.0–2.0)	0.79
Duration of constipation	0 (0–2.0)	0 (0–1.0)	0 (0–2.0)	0.81
Time spent per attempt (in minutes)	1.0 (0–1.0)	1.0 (0–1.0)	1.0 (0–1.5)	0.61
Number of unsuccessful attempts per 24 h	0 (0–1.0)	0 (0–1.0)	0 (0–1.0)	0.82
Assistance for evacuation	0 (0–1.0)	0 (0–1.0)	0 (0–1.0)	0.94
Abdominal_pain	1.0 (1.0–2.0)	1.0 (0–2.0)	1.0 (1.0–2.0)	0.05
Total constipation	7.0 (4.0–11.0)	5.5 (3.0–11.5)	7.0 (5.0–11.0)	0.29
Intestinal disorders	2.0 (0–5.0)	2.0 (1.0–4.0)	2.0 (0–5.0)	0.43
Incontinence to solid stool	0 (0–0)	0 (0–0)	0 (0–0)	0.52
Incontinence to liquid stool	0 (0–0)	0 (0–0.75)	0 (0–0)	0.85
Incontinence to gas	2.0 (1.0–3.0)	2.0 (1.0–4.0)	2.0 (1.0–3.0)	0.65
Diapers	0 (0–0)	0 (0–0)	0 (0–0)	0.62
Lifestyle alteration	0 (0–1)	0 (0–1)	0 (0–1.0)	0.36
Total incontinence	6.0 (3.0–10.0)	6.0 (4.0–9.0)	6.0 (2.0–10.5)	0.81

Bowel symptoms by sex, presented as median (interquartile range). *p*-values from Mann–Whitney U tests compare males and females. A *p*-value < 0.05 indicates statistical significance.

**Table 3:** Multiple Sclerosis Quality of Life-54 (MSQoL-54) scores.

	All	Males	Females	*p*-Value
	Median (I–III Quartile)	Median (I–III Quartile)	Median (I–III Quartile)	
Physical Function	8.5 (6.8–11.9)	7.7 (7.7–13.4)	8.5 (6.8–11.1)	0.55
Health Perception	7.6 (6.8–9.3)	7.2 (6.8–8.5)	7.7 (6.8–9.4)	0.24
Energy	4.8 (3.36–5.76)	5.3 (2.9–7.2)	4.8 (3.4–5.3)	0.47
Role Limitation Physical	0 (0–6.0)	0.0 (0.0–6.0)	0.0 (0.0–6.0)	0.50
Pain	5.1 (2.7–8.4)	5.1 (2.6–9.2)	5.1 (3.8–8.4)	0.87
Sexual Function	4.0 (2.7–8.0)	4.0 (0.0–8.0)	5.3 (2.7–8.0)	0.17
Social_Function	8.0 (6.0–10.0)	8.0 (6.0–9.7)	8.0 (5.5–10.0)	0.42
Health Distress Physical	5.5 (4.4–8.2)	5.5 (3.9–8.7)	6.1 (4.4–8.3)	0.97
Total Physical Health	43.5 (36.5–66.4)	42.7 (32.9–72.3)	48.3 (36.5–63.9)	0.58
Health distress Emotional	7.0 (5.6–11.2)	7.0 (4.9–11.0)	7.7 (5.6–10.9)	0.97
Overall Quality of Life	9.9 (8.6–12.0)	9.9 (8.6–12.2)	9.9 (8.6–12.0)	0.89
Emotional Well-Being	15.0 (3.5–18.7)	12.8 (3.5–18.6)	15.1 (6.4–18.6)	0.49
Role Limitations Emotional	0.0 (0.0–16.0)	0.0 (0.0–16.0)	0.0 (0.0–16.0)	0.50
Cognitive function	8.2 (6.0–10.5)	8.3 (6.0–12.0)	7.5 (6.0–10.1)	0.62
Total Mental Health	38.0 (27.8–64.2)	38.0 (23.5–60.8)	40.6 (27.8–64.6)	0.41

## Data Availability

The data that support the findings of this study are available on request from the corresponding author due to reasons of sensitivity.

## Abbreviations

MS: Multiple Sclerosis; RRMS: Relapsing-Remitting Multiple Sclerosis; EDSS: Expanded Disability Status Scale; QoL: Quality of Life; MoCA: Montreal Cognitive Assessment; BD: Bowel Dysfunction; WexInc: Wexner Incontinence Scale; WexCon: Wexner Constipation Scale; MSQoL-54: Multiple Sclerosis Quality of Life-54 questionnaire; PF: Physical Function; RLP: Role Limitations Physical; RLE: Role Limitations Emotional; P: Pain; EWB: Emotional Well-Being; E: Energy; HP: Health Perceptions; SF: Social Function; HD: Health Distress; OQoL: Overall Quality of Life; CF: Cognitive Function; Sex F: Sexual Function; DD: Disease Duration; FDR: False Discovery Rate; r: Correlation Coefficient (Spearman’s rank); TOT_PH: Total Physical Health Score (from MSQoL-54); TOT_MH: Total Mental Health Score (from MSQoL-54).
